# Eggspot Number and Sexual Selection in the Cichlid Fish *Astatotilapia burtoni*

**DOI:** 10.1371/journal.pone.0043695

**Published:** 2012-08-24

**Authors:** Frederico Henning, Axel Meyer

**Affiliations:** Lehrstuhl für Zoologie und Evolutionsbiologie, Department of Biology, University of Konstanz, Konstanz, Germany; Biodiversity Insitute of Ontario - University of Guelph, Canada

## Abstract

Sexual selection on male coloration is one of the main mechanisms proposed to explain the explosive speciation rates in East African cichlid fish. True eggspots are color patterns characteristic of the most species-rich lineage of cichlids, the Haplochromini, and have been suggested to be causally related to the speciation processes. Eggspots are thought to have originated by sensory exploitation and subsequently gained several roles in sexual advertisement. However, for most of these functions the evidence is equivocal. In addition, the genetic architecture of this trait still is largely unknown. We conducted bidirectional selective breeding experiments for eggspot numbers in the model cichlid, *Astatotilapia burtoni.* After two generations, low lines responded significantly, whereas the high lines did not. Body size was both phenotypically and genotypically correlated with eggspot number and showed correlated response to selection. Males with higher numbers of eggspots were found to sire larger offspring. Despite the potential to act as honest indicators of fitness, the behavioral experiments showed no evidence of a role in either intra- or inter-sexual selection. Visual-based female preference was instead explained by courtship intensity. The evolution of this trait has been interpreted in light of adaptive theories of sexual selection, however the present and published results suggest the influence of non-adaptive factors such as sensory exploitation, environmental constraints and sexual antagonism.

## Introduction

**Figure 1:**
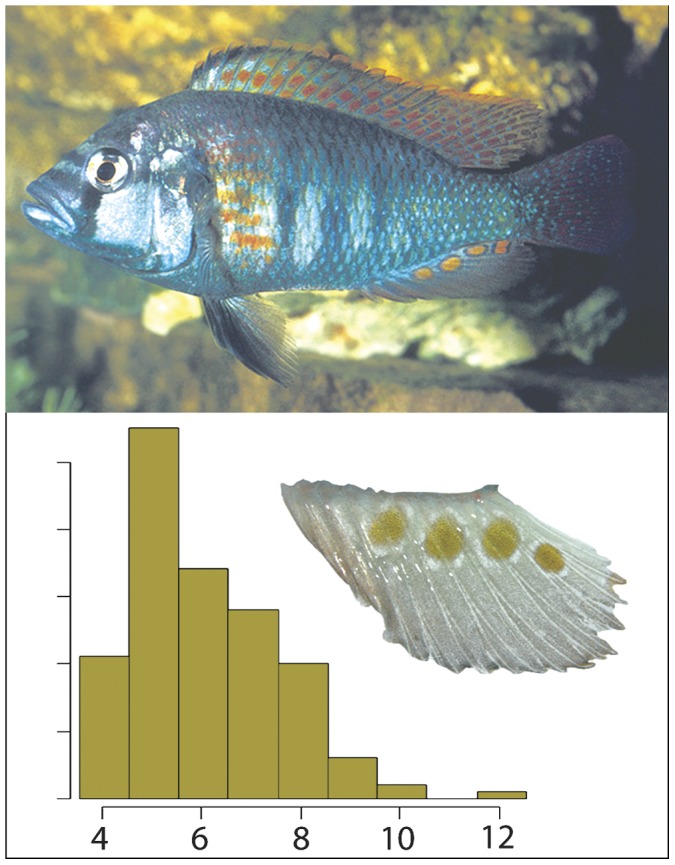
Eggspot number variation in *Astatotilapia burtoni.* A dominant male is shown on the top. A histogram showing the frequency of eggspot numbers in a sample of 167 males of 6 months of age (bottom-left). An anal fin with four conspicuous eggspots is shown on the bottom-right.

The haplochromine lineage of cichlid fishes has the fastest known speciation rate [1]. With little overall genetic differentiation, cichlids have achieved an extraordinary diversity including ecological types and coloration polymorphisms [2]. Eggspots (Figure 1), also referred to as egg-dummies or –mimics, are considered by some authors to be key-evolutionary innovations of haplochromines that might influence speciation rates [3], [4], [5], [6]. A direct role of eggspots in speciation was suggested by Goldschmidt and Visser [5], who reasoned that divergent selection regimes on egg morphology could lead to divergence of eggspots and female preference, thus facilitating speciation. Furthermore, the origin of this trait coincides with the origin of the modern haplochromines leading to the perception that eggspots might be causally involved in the explosive speciation rate of this lineage [3]. In the present manuscript, we analyze the short-term response of number of eggspots to artificial selection and test the hypotheses that the number of eggspots is an honest indicator of survival and social status and therefore a target for adaptive female choice.

Eggspots are color traits usually located on the anal fin and are considered to mimic eggs. They play many important roles in the mating behavior of haplochromines. In order to attract mates, territorial males approach and quiver their anal fin thereby displaying it and their eggspots to females. During courtship and mating displays, both sexes perform this quivering behavior and this induces the partner to mouth at their anal fin, eliciting spawning and release of semen (see Video S1). Female haplochromines collect the eggs in their mouth immediately after they are spawned, presumably as an adaptive response to the heavy rates of predation on eggs. It was originally proposed that eggspots deceive females to mouth at the males anal fin to ensure fertilization [4]. However, the experimental removal of eggspots had no effect on fertilization success [7], [8]. Some mate choice studies suggest that advantages to males can instead derive from increased mating frequency [7]. It was reported that in several haplochromines, females discriminate among males based on presence [7], particular number and size [9] and high numbers [7], [8], [10]. Most haplochromines have a variable numbers of eggspots. In others, such as *Pseudotropheus* (*Maylandia*) *lombardoi*, it was reported that males have a single spot and that female mate choice is the likely source of selection [9].

By mimicking eggs eggspots might constitute one of the few clear examples of sensory exploitation and pre-existing bias [11] and might have evolved by exploitation of an ancestral sensory bias [12]. Empirical and theoretical studies suggest that sensory exploitation might be important for the initial evolution of preference and signal [13]. The costs of being exploited by males can subsequently favor the evolution correlations with fitness-related traits and sexual advertisement functions leading to adaptive female choice [13]. A role in sexual advertisement was suggested to account for female preference for high numbers of eggspots. It was proposed that high numbers indicate genetic quality [8] and are correlated with male fitness traits such as survival and social dominance [14].

Little is known about the factors responsible for the variation in numbers of eggspots. Even the simplest explanation for the variation in numbers (positive phenotypic correlation with body size) has received mixed support [14], [15]. Furthermore, the sources of sexual selection (both inter- and intra-sexual) are based on a limited number of studies and species and modest sample sizes [7], [8], [9], [10], [14].

In spite the enthusiasm for eggspots and their role in cichlid speciation, the genetic architecture and transmission genetics of this trait still is largely unknown. Artificial selection experiments are invaluable tools for directly investigating the response to selection, general evolvability (sustainability of the response) and the existence of genetic correlations [16], [17]. Few studies have applied these methods to teleosts models of evolutionary and not aquacultural research [18], [19], [20].

The focal species of the present study, the model haplochromine *Astatotilapia burtoni* is polymorphic for eggspot numbers and exhibits lek-like mating behavior [21]. A recent study on the variation of eggspot numbers in this species shows that numbers of eggspots are highly heritable. On the basis of a correlation with age and success in male-male competition, the authors proposed that high numbers of eggspots function as sexual advertisers [14].

The present study aims to analyze a) the response of eggspot numbers to bidirectional selection b) the phenotypic and genetic correlations with body size; c) the strength of female mating preference on this trait and; d) test the sexual advertisement functions. In addition, ontogenetic series were analyzed to disentangle of effects of aging and growth on eggspot numbers and assess the proximal mechanisms of eggspot gain.

## Materials and Methods

Experiments were approved by the German authorities (Regierungspräsdium Freiburg, Abteilung Landwirtschaft, Ländlicher Raum, Veterinär- und Lebensmittelwesen).

### Response to Artificial Selection

Bidirectional artificial selection for eggspot number was carried out for two generations using a stock of *Astatotilapia burtoni* maintained and randomly mated in the laboratory for over 10 years (generation time is about six months). Three selection lines (low, high and control), with two replicates each, were established from a base population of 82 individuals (58 males and 22 females) of approximately one year of age. The distribution of eggspot number of the base population is shown in Figure S1. First, six individuals were randomly selected to constitute each of the control lines then the three males and females with the highest and lowest numbers of eggspots were selected to establish the first generation of the high and low lines, respectively. To avoid sib matings, the second generation was generated by selecting one male and one female from each family (within-family truncation selection). Each replicate was housed in a single tank, and the families kept separate using mesh dividers. To avoid effects of density resulting from different brood sizes, families were reduced three months prior to selection to make their sizes comparable to the smallest family. The tanks were randomized in each generation of selection. At six months of age, pairs were selected, isolated and kept separate using plexi-glass (to avoid aggression from the male and ensure visual contact and maintenance of an active reproductive state). The pairs were inspected daily and allowed to interact without the dividers after showing signs of sexual behaviors. In these conditions, all males become territorial and reproductively active and most females spawn in regular intervals (20–30 days). Ripe females have a distinct morphology (swollen abdomen and genital papilla) and respond actively to male courtship. The sample sizes are shown in Table S1. In one case (control line, replicate one) a brood consisted exclusively of females. Although females normally spawn in regular intervals, in some cases the selected females failed to spawn or the pair failed to mate successfully. These factors caused the second generation to consist of one (low 1, control 1 and high 2) or two families (high 1 and low 2) in some lines. This does not affect our analysis since inbreeding is not biased towards a particular selection line or phenotypic value.

Because the eggspots of females are sometimes difficult to count unambiguously, only the male values were used in the analysis of selection response. The number of eggspots was counted based on standard photographs and scaled to the mean and S.D of each sex/replicate (z score). The significance of the response to selection of eggspots and correlated response of body size was analyzed by comparing selection to control lines using Mann-Whitney tests because normality of the data in all replicates and lines was rejected (Shapiro-Wilks test, *P*<0.05).

Heritability was estimated using the animal model (mixed model) approach according to Wilson et al. [22] using the MCMCGlmm R package [23]. This estimate was based on the total pedigree information and all the phenotypic records. The mixed model approach including females and was chosen instead of the traditional midparent-offspring correlation because it is more robust to the violations of assumptions of absence of selection and normality. This method can also account for different underlying error distributions, as is expected to be the case with sex in the present study (female values are uncertain, particularly at lower phenotypic values) [16], [24]. Models were compared using the Akaike and deviance information criteria (AIC and DIC). The final model had sex as a fixed effect and tank, selection line, age and animal (pedigree matrix) as random effects. To test for genetic correlations between eggspot number and body size, offspring eggspot number and body length were regressed on midparent body length and eggspot number, respectively.

### Female Mating Preference

Association time, a widely used predictor of mating preference [10], [25], [26], was measured as a proxy for female preference. The test tank (500 l, 190×70×40 cm) was divided into three enclosures using glass dividers. The two most extreme quartiles of the center compartment were considered choice zones. The time spent in each of the three zones was recorded for 20 min. All trials were video-taped for later quantification of male courtship. One male from each group was introduced randomly to each of the lateral compartments (125 l, 50 cm length). Males were acclimated for at least two hours in the presence of con-specifics before the trial.

37 mature males (13–18 months of age) were selected and paired to minimize size difference and maximize the difference in eggspot numbers. Two groups consisting of males with high (9–13, n = 8) and low (6–7, n = 8) numbers of eggspots were used in the valid trials. To achieve a good match of size, weight and eggspot number some males had to be used more than once, but were paired to different males. The mean difference in eggspot number between the two males in each trial was 5.09 (S.D. = 1.87). All males were kept in individual tanks for at least four days prior to the experiment. A ripe female (identified visually from a stock of 26 kept in sexual isolation) was transferred to the center compartment immediately before each trial. Females took less time to acclimate than males, and were actively exploring the test tank one or two minutes after being introduced.

Courtship intensity was measured by summing the counts of the following male behaviors: *approach, quiver* and *lead swim*. The sum was normalized by the time spent in proximity to the female to avoid obtaining a spurious correlation. Males only court in the presence of females, so the male with more association time automatically courts more. After the trial, the test female was transferred to a tank containing a sexually active, territorial male to determine whether the female was ripe. Ripe *A. burtoni* females mate immediately with the resident male in these conditions. Some can take up to 48 h depending on the degree of maturation of the eggs.

Mate choice trials were considered valid when a) both males courted; b) the female had visual contact with both males within the first 10 min and c) the female spawned within 48 h of the trial [10], [25]. Multiple-regression was carried out using female association time as the dependent variable and factors known to influence mate choice in haplochromines were independent variables. Those were pelvic fin length, standard length, eggspot number and courtship intensity. The values included in the analysis were calculated as the difference between both males. Model simplification proceeded through sequential dropping of the least significant terms. Nested models were compared using F tests. All statistical analyses were performed using R version 2.10.1 [27].

### Phenotypic Correlation with Body Size and Ontogeny

To test whether eggspot numbers can act as an honest indicator of survival, we investigated the correlation of eggspot number (response) with standard length and age (predictor variables) on a sample of 542 males. Colinearity of the predictors was assessed by calculating correlation coefficients and the variance inflation factor (VIF) using the R package MASS. The analysis was initially carried out using a generalized linear model (GLM) with Poisson errors. However the residuals were not normally distributed, there was evidence of heteroscedasticity and very high VIF values. The data was further analyzed in a linear model (LM) with robust estimates of the standard error and *P* values obtained using a sandwich covariance matrix estimator in the R package *sandwich* [28]. In a second LM, the response variable was Box-Cox transformed based on the likelihoods of the λ values using the R package MASS and analyzed in a LM. The transformation led to a more reliable model in which errors were normally distributed and homoscedastic. To disentangle the unique contributions of age and size, a residual regression was performed. In this approach, the residuals of the regression of weakest predictor on the strongest are used instead of the observed values [29].

The mechanisms of eggspot addition were investigated through the analysis of ontogenetic series from 14 individuals. All specimens were raised in individual tanks to avoid potential effects of social environments and photographed at approximately 6 months of age and in intervals of 2–6 months until the age of 24 months. Ontogeny of eggspots in earlier stages has already been investigated [30].

### Male-male Competition

The correlation of eggspot number with dominance was assessed by allowing two males to directly compete in a tank measuring 90×70×40 cm (250 l) in the presence of 10 females (added to improve acclimation). Under these conditions, males engage in intense territorial battles within 10 min. A total of 112 males were photographed and paired to minimize size difference and distribute other factors (such as age and social context) uniformly between the two groups of males with high and low numbers of eggspots. 33 pairs (total number of males  = 66) were selected. The difference between both males in relevant traits is shown in supporting Figure S2.

Subordinate males were immediately removed after the hierarchy had been established, which usually took place within 5–20 min. The probability of success of the individuals with more eggspots in the 33 trials was tested against the null hypothesis of 0.5 using a binomial test.

## Results

### Response to Artificial Selection

**Figure 2:**
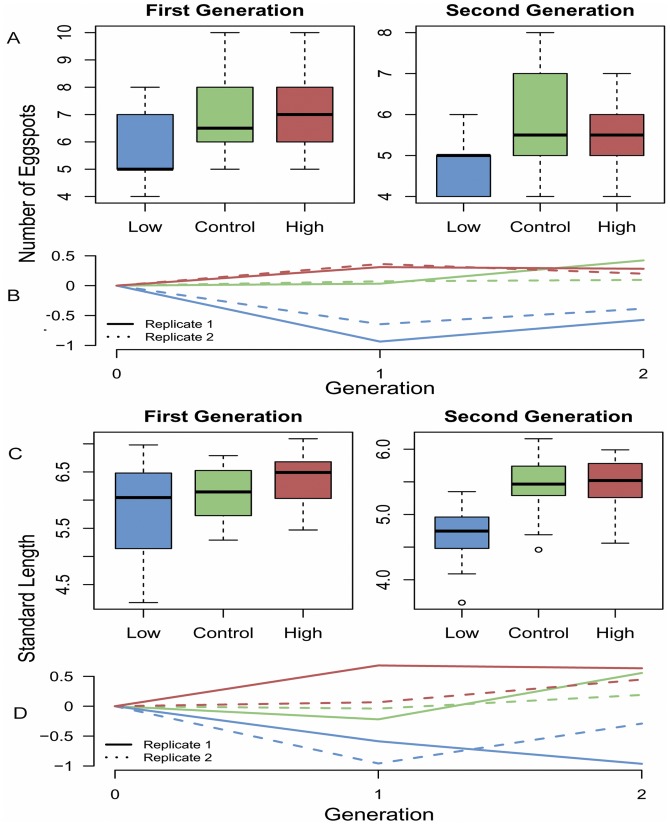
Response and correlated response to selection. Response of eggspot numbers (**A**,**B**) and correlated response of body size (**C**,**D**) to two generations of artificial selection. (**A**) Boxplots of eggspot numbers of each selection line. (**B**) Selection response trajectories. Eggspot numbers were scaled (z score) to eliminate the difference in range between the generations. Plotted values are the means of each replicate. (**C**) Boxplots of standard length of each line. (**D**) Correlated response trajectories of standard length values scaled (z score) to eliminate the difference in range between the generations and sexes. Values plotted in the line plots (**B** and **D**) are the means of each replicate.

The standardized selection differential and responses are given in Table 1. The response to selection was asymmetric, the low line responded significantly whereas the high line did not (Table 2, Figure 2A). The response trajectories are shown in Figure 2B. The number of eggspots of the low line differed significantly from the control line in both the first generations. Due to biased sex ratios and failure to spawn in one family (see Materials and Methods), replicate 2 of the low line (generation 2) consisted of only 2 males. In this case, the small sample size did not allow for statistical significance although the trend is clear (Figure 2B). The high line was not significantly different in any of the generations. The narrow sense heritability estimated with the animal model was of 0.38±0.2.

**Table 1:** Standardized selection differentials (*S*) and response (*R*) for each of the selection lines.

Generation	Line	Replicate	*S*	*R*
1	Control	1	0.12	0.07
		2	−0.36	0.03
	High	1	2.19	0.36
		2	1.87	0.31
	Low	1	−1.32	−0.65
		2	−1.16	−0.94
2	Control	1	1.85	0.1
		2	−0.15	0.42
	High	1	0.35	0.3
		2	0.35	0.28
	Low	1	−1.89	−0.39
		2	−1.52	−0.48

Values are presented as z scores (as described in the Materials and Methods section).

**Table 2:** Results of two-sample Wilcox test of response to selection.

Line	Generation	Replicate	*W*	*P* value	*N*
Low	1	1	205.5	**0.027**	15
		2	244.5	**0.016**	16
		Combined	887	**0.002**	31
	2	1	3	0.617	2
		2	509	**<0.001**	28
		Combined	590	**<0.001**	30
High	1	1	125	0.546	15
		2	132	0.612	14
		Combined	499.5	0.3187	29
	2	1	16.5	1	17
		2	163.5	0.813	13
		Combined	415	0.672	30

The test statistic (*W*), *P* values and number of males in each replicate are given (*N*). Significant *P* values are shown in bold.

The correlated response of body size is shown in Figure 2C. Body size showed significant response in the first generation of selection for high numbers of eggspots (W = 1062.5, *P* = 0.012) and for low numbers in the second generation (W = 2773, *P*<0.001) (Figure 2D).

### Female Mating Preference

24 trials were conducted, out of which 10 were considered valid according to the criteria outlined in the Materials and Methods section. Seven trials were eliminated due to criteria *a*, two due to *b* and five due to *c.* Females that were not ripe spent more time in the neutral zone (48%) than females from valid trials (16.7%). This indicates that the difference in association time between males reflects sexual choice, rather than schooling behavior. Some examples of the recorded mating behaviors are shown in the Video S1.

**Figure 3:**
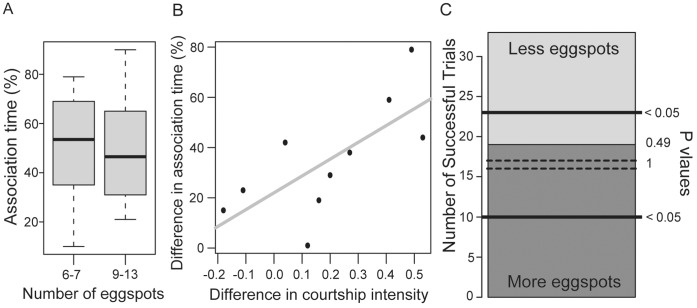
Results of behavioral trials. (**A**) Association times of the two groups of males with high and low numbers of eggspots. Boxes represent the interquartile range, the median is shown by the dark bar and outliers are displayed as circles; (**B**) Regression of association time on the difference in courtship intensity of the two males in each trial; (**C**) Number of trials won by males with lower (light grey) and higher (dark grey) numbers of eggspots is shown on the stacked barplot. The left axis shows the total number of trials (33) and the height of each stack represents the number of trials won. The right axis shows the associated significance values for a binomial test with 33 events. Values more extreme than the two full lines (>22 or <11) are significantly different from a 50% chance of winning.

Female association time was normally distributed (Shapiro-Wilks test, W = 0.975, *P* = 0.932) and was not significantly correlated with pelvic fin length, body size or eggspot number (Figure 3A). Courtship intensity was significantly correlated with female association (adjusted *r^2^* = 0.435, *F* = 7.941, 8 df, *P = *0.023) (Figure 3B).

### Male-male Competition

All pairs of males engaged in aggressive territorial disputes and the dominance hierarchy was clearly established in all trials. The males with higher numbers of eggspots were dominant in 19 out of 33 trials. The estimated probability of success of 0.58 (95% C.I., 0.39–0.75) does not differ significantly from 0.5 (binomial test, *P* = 0.49) (Figure 3C).

### Phenotypic Correlation with Body Size and Ontogeny

Standard length had a significant positive effect on eggspot numbers in all analyses. Those were a GLM (β = 0.17, S.E. = 0.013, Z = 13.77, *P*<0.001), a LM with robust estimates (β = 1.08, S.E. = 0.055, T = 19.72, *P*<0.001) and a LM with transformation of the response variable (β = 0.03, S.E. = 0.001, T = 28.84, *P*<0.001). The effect of age was not significant, and dropped from the models.

**Figure 4:**
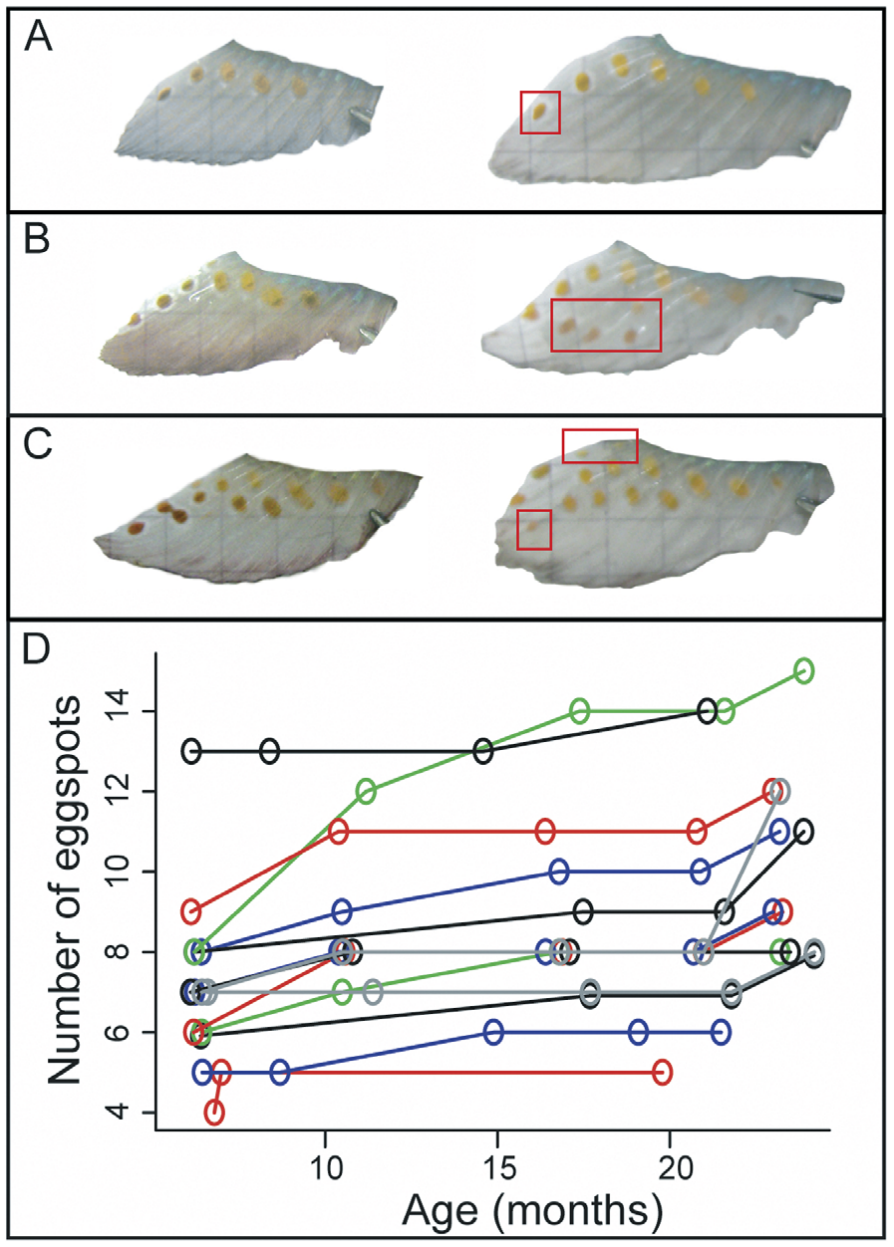
Ontogeny of eggspots. Formation of eggspots of three specimens on the tip (**A**), center (**B**) and proximal (**C**) regions of the anal fin are shown. Pictures on the right are from the same individual at a later time point. New eggspots are highlighted in red boxes. The ontogenetic trajectories of all 14 individuals (each represented by a line) are shown in **D**.

The formation of 32 eggspots in 14 individuals was observed in the ontogenetic series. All individuals gained eggspots at the tip of the anal fin (mean number of new eggspots per individual  = 1.57) (Figure 4A), three individuals (21%) gained eggspots at the proximal region (Figure 4B) and two individuals (14%) gained eggspots at the center of the anal fin (Figure 4C). The ontogenetic trajectories of all 14 individuals are shown in Figure 4D.

## Discussion

Here we show that a) eggspot number responds significantly and asymmetrically to bidirectional selection with correlated response of body size; b) the phenotypic and genetic correlations with body size are strong, and likely due to the ontogenetic mechanism by which eggspots are formed and; c) the main visual cue for mate choice in this species might be courting intensity. Our results, based on larger samples, broader distribution of the predictor variables (age and size) and more appropriate experimental designs, question the strength and universality of the previously proposed sources of sexual selection and the notion of a paradox of standing genetic variation in this trait. Instead, we propose that the current levels of standing additive genetic variation are consistent with the existence of weak selection pressures and possibly of selection on a genetically correlated and condition-dependent trait.

### Response to Artificial Selection

**Figure 5:**
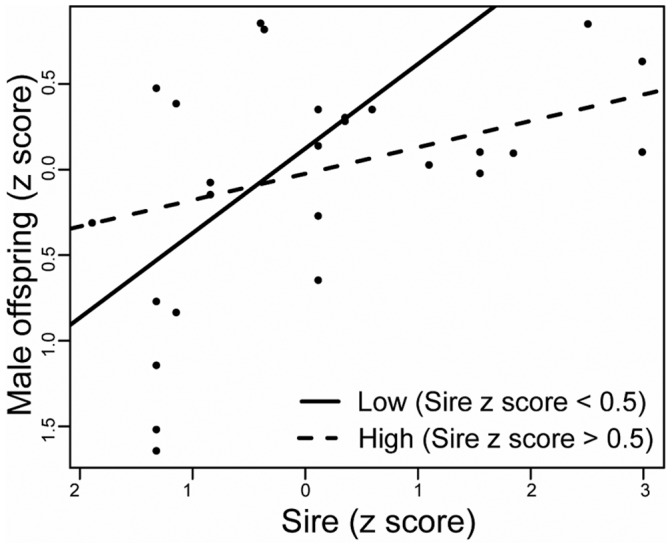
Realized heritability. Scaled eggspot numbers of male offspring are regressed on sire values. The regression lines for sires with phenotypic values of less and more than 0.5 are shown to illustrate the asymmetric response to selection.

We found an asymmetric response to selection, the lower half of the distribution of eggspot numbers (z <0.5) had nearly double the realized heritability of the upper half (Figure 5). Asymmetric responses to selection are a common finding in selection experiments. The reasons given by Falconer and Mackay [31] involve experimental artifacts (random drift,inbreeding depression and unmeasured natural or sexual selection acting during the experiment) as well as a multitude of potential genetic causes (genetic asymmetry, presence of major genes, scalar asymmetry). Random drift is unlikely to explain the present results because of the consistency of response between both replicates (Figure 2B). Inbreeding depression is also unlikely because the mean of the unselected control line did not decline. Post-hatching mortality was negligible in the present experiment, thus indicating that differences in viability also cannot account for the asymmetric response. This suggests that the observed asymmetry is likely to have a genetic cause. Wild caught *A. burtoni* specimens hardly develop more than a single row of eggspots (G. Fryer, personal communication). But wild caught broods reared in the laboratory for a period of one year also show high range of variation and multiple rows (F. Henning, personal observation). Although we can not at this point rule out the other genetic causes (major genes, directional dominance or genetic asymmetry), it is possible that the asymmetric response is explained by scalar asymmetry: high phenotypic values might be particularly subject to environmental influence (e.g condition-dependency, rearing conditions) and the extreme values an artifact of laboratory rearing. The absence of individuals with phenotypic values above 0.35 (see Table 1) in the first generation of the high line also suggests that the more extreme high values observed in the base population have non-genetic causes and supports the scalar asymmetry hypothesis. The intensity of selection that could be applied on the second generation in the high line (Table 1) was constrained by two factors: The lack of response of the first round of selection and the smaller variance of eggspot numbers in the first and second generations (6 months of age) as compared to the base population (one year of age).

One interesting possibility is that the asymmetry results from the previous action of selection in the base population. Favorable alleles are expected to have frequencies above their symmetrical points [31]. This would suggest that the low numbers of eggspots (<6–7) or small body size were previously selected against.

Body size responded in the direction of selection for eggspot numbers in two cases (high line in generation one and low line in generation 2). Although this pattern is not as clear as the direct selection response (presumably due to environmental variance), cross-heritability is high (0.60±0.26) and significant when estimated by the regression of mean male offspring body size on sire eggspot number (*r^2^* = 0.17, *F*_1,24_ = 6.021, df, *P* = 0.02). Due to the advantages that body size confers to males in the competition for social dominance, it would be expected that females use eggspot number as a mate choice cue.

### Do Females have Strong Preferences for Eggspot Numbers?

Female association in *A. burtoni* was predicted by courtship intensity with little or no effect of eggspot number. Preference for males that court in high frequency is widespread, but not necessarily open-ended [32]. Activity level is a potential sexual advertiser, as it can be an indicator of condition and viability [33]. Despite the limited sample size (in part due to the stringent validation criteria that were used), the significant association with courtship intensity and the difference in association time between mature and immature females shows that our experiment was valid and had enough power to detect the variable that best explained female preference.

Sexual selection might instead act on eggspot numbers by influencing the size of clutches that are spawned. The number of clutches spawned with males more eggspots was larger in other cichlids, *A. elegans* [7] and in *P. aurora* [8]).

A recent study on *A. burtoni* [34] could not demonstrate a statistically significant effect of the number of eggspots on the male’s mating success. Only one replicate (out of three) was significant in one experiment. Furthermore, all females in each of three replicates chose among the same four males. The finding, that one single male with no eggspots was more successful might well have been due to other causes (perhaps activity level) [33]. For these reasons, we have to regard the evidence for the effect of eggspot numbers on female choice in *A. burtoni* as tentative only.

### Does Eggspot Number Signal Social Status?

Eggspots have also been seen as a signal for social dominance and, in this way, are related to spawning success [14], [34]. But even with our larger sample, we did not detect a significant relationship between eggspot number and intra-sexual competition. We believe that the discrepancy might be best explained by the differences in the type of statistical analyses and sample sizes used in both of the previous studies on this issue.

Lehtonen and Meyer [14] used two different experimental set-ups to investigate the correlation of number of eggspots with male dominance. In the first approach, no significant differences were found. The second experiment (where an effect was detected), was similar in design to the one we chose in this present study but the analysis was carried out by comparing the mean number of eggspots of the successful and unsuccessful males using a paired T-test. In the present study, more trials were analyzed and we opted to treat success in the competition (instead of eggspot number) as the response variable. The effects reported in Lehtonen and Meyer [14] are also not significant when analyzed with a binomial test.

Theis et al. [34] recently tested whether males’ aggression preference is influenced by their opponents’ eggspot numbers and a marginally significant effect was reported [34]. Details of the GLMM analysis were not given but the reported results are not significantly different from a 0.5 probability of success using a binomial test. Although the response variable was number of attacks, the data was coded as binary and we believe that a normalized measure of the difference in attacks received by both males would have made better use of the data.

Based on the current results and the published evidence, we conclude that evidence for a role of eggspot numbers in male-male competition is also tentative. It is possible that the number of egg-like pigmentation patterns are associated with intra-sexual competition by having a signaling function or by pleiotropy with aggressiveness. However, a lack of association can hardly be considered surprising given that the primary function of eggspots is in inter-sexual competition.

### Does Eggspot Number Signal Survival Capacity?

Previous studies yielded contradictory evidence regarding the relationship of eggspot number with body size and age. A positive correlation was found in one study, but the confounding effect of age was not controlled for [15]. In another study it was reported that the two traits correlate in young (7.5 months) but not in older adults (10 and 16 months) [14]. The authors argue that the stronger effect of age indicates that eggspot number is an honest indicator of survival capacity. However, the sample sizes were limited and the significant positive correlation was found in the age group with the largest sample [14]. In the same study, an experiment aimed to investigate the costs of producing of eggspots by analyzing whether this is a condition-dependent trait. A significant difference between the treatments (food restricted vs. control) was found, but the inclusion of body size in the GLM analysis rendered the treatment effect insignificant. This indicates that it was the differential growth in the good-condition treatment that led to a difference in eggspot number. Besides using a larger sample, we concomitantly estimated the effects of aging and body size on eggspot number using the full range of these variables in natural populations [14], [35].

The data on the ontogenetic series presented here shows that eggspots are most commonly formed at the tip of the anal fin. This process also explains why body size predicts eggspot number better than age. The initial three to five eggspots are formed at the center of the fin before individuals reach the size of 25 mm [30] and the sequential adding of eggspots at the tip of the fin (probably responsible for most variation in eggspot number) is correlated with growth rate. Apart from terminal addition, we have observed three instances of eggspot formation in the centre of the fin. Size is one of the main determiners of dominance in cichlids and since only dominant males hold territories and breed, eggspot numbers can show correlated response to selection on size, as was the case in our selection experiment.

One standing dilemma in the sexual selection literature, known as the *lek paradox*, is the persistence of female preference despite the depletion of additive genetic variation in male traits caused by sexual selection [36]. The heritability of eggspot number in our laboratory stock is high (0.5±0.2 in Lehtonen and Meyer [14] and 0.38±0.02 in the present study) and this was considered paradoxal [14]. Our results show however that sexual selection for eggspot number through female choice is weak or absent and provide a simple explanation for the persistence of highly heritable variation. The high estimates of heritability are consistent with a trait under weak selection. By failing to support the basic assumption of strong mate choice, the present and previously published reports [34] are inconsistent with the existence of a paradox of standing genetic variation on this trait. The results presented here support the existence of an indirect source of selection on eggspot numbers. Such indirect selection allows the persistence of additive genetic variation since the trait under direct selection (body size) is highly condition-dependent [37], [38].

Field studies reported that *A. burtoni* inhabits mainly river deltas and highly turbid shore ponds surrounding Lake Tanganyika [21], [39]. In particular, the murky ponds are considered a protective breeding habitat [21], [39]. Based on our own observations, that would be the case in at least many habitats of this species. The authors state that the turbidity of the shore pools “*might be of prime importance in the behavioral interactions*” [39]. We also found these fish in the Kalambo River flowing into Lake Tanganyika. That river varies strongly in its water clarity depending on the season and the location in the river (AM pers. obs.). It is plausible that turbidity impedes female discrimination and has constrained the evolution or maintenance of female preference for eggspot number. Turbidity impairs visual discrimination and is known to affect and constrain visual-based sexual selection [40], [41], [42]. This can also have led to a shift from visual mating preference to behavioral cues such as courtship intensity or to acoustic signals which were recently suggested to play a role in *A. burtoni* courtship and preference [43].

### Final Remarks

The asymmetric response to artificial selection can be explained by scalar asymmetry (where high phenotypic values have non-genetic causes) or previous action of sexual selection in the base population. The strong effect that body size has on social status and hence reproductive success suggests that the target of selection is unlikely to be the number of eggspots but rather a correlated trait.

Body size is one of the main determiners of social status and consequently, reproductive success in haplochromine cichlids [44]. Because of the genetic correlation, eggspot number is expected to be maintained at high numbers because of intra-sexual selection, even in the absence of female preference or a direct role in male-male competition. This might in fact explain the asymmetric selection response. It is unlikely that sexual selection shifts gene frequencies from their point of equilibrium, but male-male competition based on size might. The intensity of the stimulus associated with the presence of more eggspots provides a more straightforward measure of relative size since males do not necessarily form adjacent territories or compete directly for mates. Considering that the cross heritability between both traits is high and males with more eggspots tend to sire larger sons, it would be expected that females use eggspot numbers as a signal of genetic quality [45]. That they do not seem to do so might be explained by constraints on visual discrimination associated with turbid waters [21,39,but see 46] or sexually antagonistic effects [47]. Anecdotal evidence (high frequency of non-spawning, large females in the high line) suggests that males with high numbers may sire non-sexy daughters. If this observation were upheld in future investigations, a potential explanation could be the incomplete resolution of sexual conflict (this species does not posses heteromorphic sex chromosomes).

Despite suggestions in the literature that eggspot number might be an important trait for adaptive mate choice [8], [9], [10] and speciation by sexual selection [3], [5], [6], we found little support for this hypothesis in our mating preference trials. If there is indeed female preference for eggspot numbers in *A. burtoni*, it is weaker than in other haplochromines, not the primary mate choice cue and likely to be overcome by male-male interactions.

We also found no support for the hypothesis that eggspots gained sexual advertisement functions by signaling survival capacity and social status [7], [14]. It is possible, that eggspots are maintained purely as exploiters of female biases and this should be explored by future experiments.

It remains to be seen whether these results can be extrapolated to more species. Using experimental designs and sample sizes similar to the ones used here, female preference for eggspot numbers was reported in two Malawian cichlids, *Pseudotropheus (Maylandia) lombardoi* and *P. zebra* [9], [10]. However, a cursory examination of the overlap of eggspot numbers from closely related species undermines the role of this trait in species recognition (see Table S2 for a compilation of eggspot numbers taken from popular cichlid books). For example, most species of the genus *Pseudotropheus* (*Maylandia*) have between one and six eggspots [see pictures in 48 and Supporting Figure S3]. Considering this substantial overlap, discrimination based on this trait would only be effective for few species pairs. The same is true for the other African great lakes [46], [49]. We stress that the present paper dealt with only one (number) of the many eggspot related traits (size, distribution, color) and perhaps these other traits are more important for species recognition [5], or simply the role of this structure in speciation might have been overstated.

## Supporting Information

Figure S1 **Histogram of eggspot numbers in the base population consisting of 82 individuals (58 males and 22 females) of one year of age.**
(TIF)

Figure S2 **Differences between males in each male-male competition trial.** The only trait that differs systematically between the two groups is eggspot number.
(TIF)

Figure S3 **Boxplots of number of eggspots of the Malawi genus** ***Maylandia*** **compiled from Konings (2007).**
(TIF)

Table S1 **Numbers of individuals of each selection line.**
(XLS)

Table S2 **Numbers of eggspots of several species of haplochromines compiled from popular cichlid literature.**
(XLS)

Video S1 ***Astatotilapia burtoni*** **mating behavior.** Examples of behaviors observed during mating preference trials are shown.
(M4V)
