# Supplementary figures and images for: Eggspot Number and Sexual Selection in the Cichlid Fish Astatotilapia burtoni
Source: PLoS One. 2012 Aug 24;7(8):e43695. doi: 10.1371/journal.pone.0043695 (PMC3427294; doi:10.1371/journal.pone.0043695)

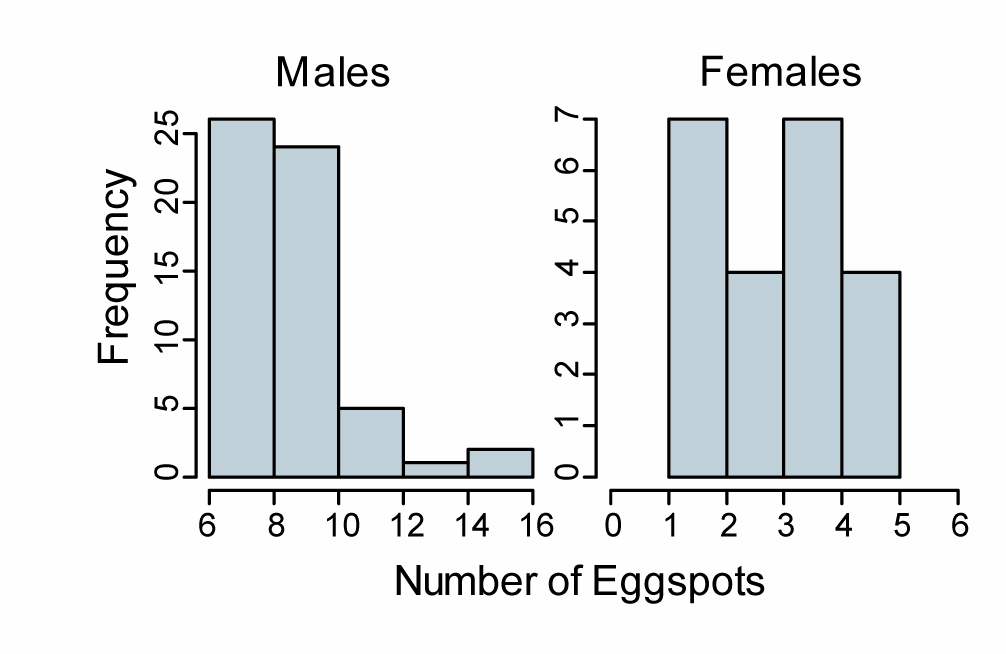

Supplement: Figure S1 — Histogram of eggspot numbers in the base population consisting of 82 individuals (58 males and 22 females) of one year of age. (TIF) [file pone.0043695.s001.tif]

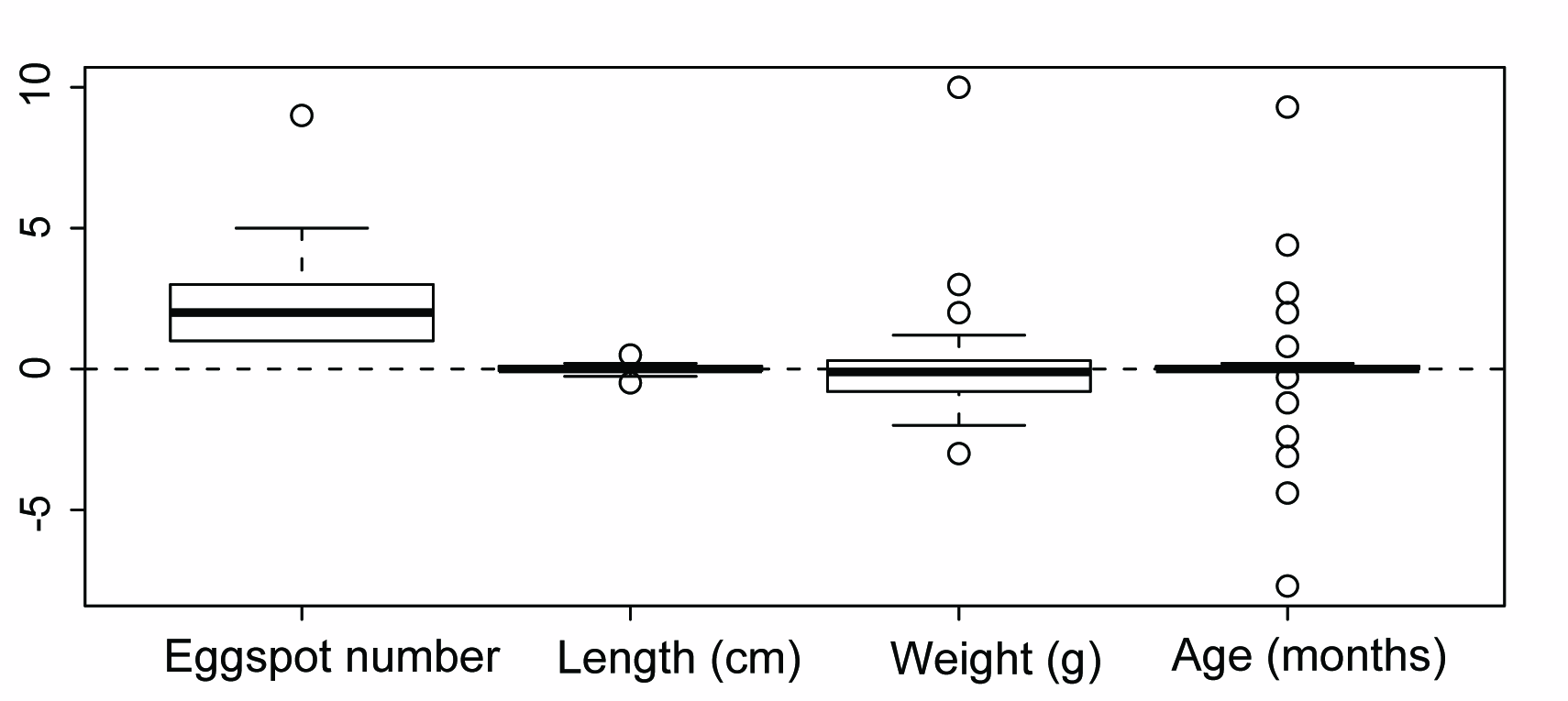

Supplement: Figure S2 — Differences between males in each male-male competition trial. The only trait that differs systematically between the two groups is eggspot number. (TIF) [file pone.0043695.s002.tif]

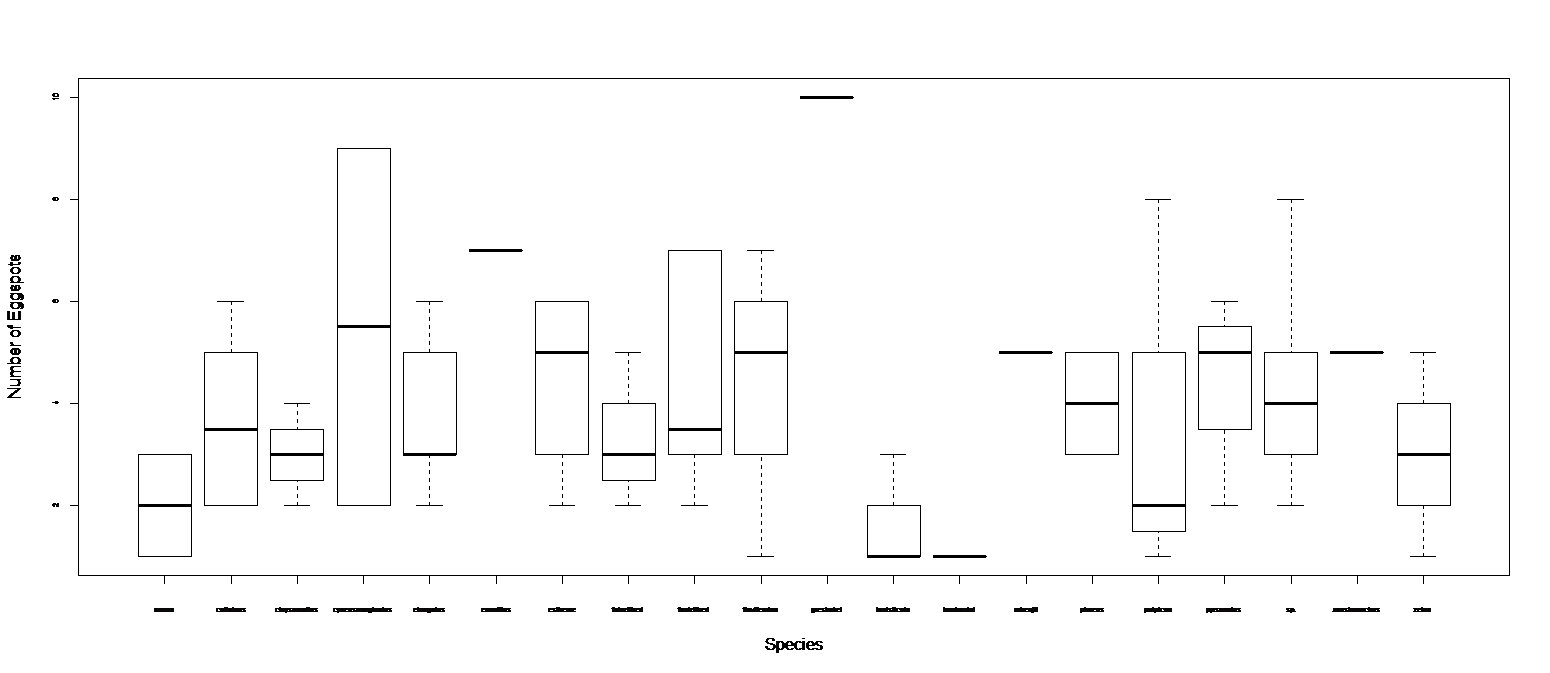

Supplement: Figure S3 — Boxplots of number of eggspots of the Malawi genus Maylandia compiled from Konings (2007). (TIF) [file pone.0043695.s003.tif]
